# Endoscopic endonasal management of papillary and adamantinomatous craniopharyngiomas: a comparative operative video with a proposed role for intraoperative Raman spectroscopy

**DOI:** 10.3171/2026.7.FOCVID26127

**Published:** 2026-10-01

**Authors:** Nesrine Jemel, Ana Bokasova, Najib Nassiri, Philippe Lavigne, Frederic Leblond, Moujahed Labidi

**Affiliations:** 1 Division of Neurosurgery, Centre Hospitalier de L’Université de Montréal (CHUM), Québec;; 2 Lumedlab, Polytechnique Montréal;; 3 Centre de recherche du CHUM (CRCHUM), Montréal; and; 4 Division of Otolaryngology–Head and Neck Surgery, Centre Hospitalier de L’Université de Montréal (CHUM), Québec, Canada

**Keywords:** papillary craniopharyngioma, adamantinomatous craniopharyngioma, endoscopic endonasal, Raman spectroscopy

## Abstract

Craniopharyngiomas comprise two molecularly distinct subtypes: papillary craniopharyngioma (PCP), driven by BRAF V600E mutations, typically presenting as solid, well-circumscribed third ventricular masses in adults; and adamantinomatous craniopharyngioma (ACP), driven by CTNNB1 mutations, featuring cystic-solid architecture with calcifications and hypothalamic adherences. Two representative cases illustrate their radiological and intraoperative differences via the extended endoscopic endonasal approach. Subtype identification through MRI, intraoperative frozen-section analysis, and CSF liquid biopsy is critical, as it directly shapes surgical strategy and postoperative decisions, including BRAF/MEK inhibitor therapy for PCP. Raman spectroscopy holds promise as a future real-time intraoperative discrimination tool.

The video can be found here: https://stream.cadmore.media/r10.3171/2026.7.FOCVID26127

## Transcript

This video is a comparative operative video of endoscopic endonasal management of papillary and adamantinomatous craniopharyngiomas with a proposed role for intraoperative Raman spectroscopy.

### 0:31 Clinical Presentation—Case 1: Papillary Craniopharyngioma.

Our first case with papillary craniopharyngioma is a 69-year-old male who presented with progressive binocular visual disturbance and no headaches. Neurological exam revealed reduced visual acuity and bitemporal hemianopsia. Preoperative endocrine assessment was normal.

### 0:48 Neuroimaging Findings—Case 1: Papillary Craniopharyngioma.

Imaging revealed a suprasellar mass with a round, cystic, berry-like papillomatous surface. Computed tomography demonstrated calcifications. On T1, the lesion appeared hypointense with homogeneous gadolinium enhancement. Notably, the pituitary stalk was intact and wholly visible below the tumor.

### 1:05 Surgical Video—Case 1: Papillary Craniopharyngioma.

Drilling proceeds sequentially along the sellar floor, tuberculum sellae, and planum sphenoidale, achieving wide exposure from the sella turcica to the chiasmatic cistern. Following dural opening, the operative field reveals a clear color distinction between the yellow hue of the normal pituitary gland and the gray appearance of the suprasellar tumor, facilitating precise identification of the tumor-gland interface.

Dissection is carried out along the tumor margins, progressively developing the arachnoid plane to preserve the perforating vessels and the pituitary stalk. The resection alternates between internal debulking and sharp/smooth capsule dissection, allowing progressive mobilization of the tumor while maintaining critical neurovascular structures. A portion of adherent capsule is identified at the junction of the infundibulum and optic chiasm and is left in place.

Reconstruction is performed using a multilayer technique: a collagen matrix inlay is placed, followed by autologous fascia lata overlay. Finally, a vascularized nasoseptal flap is used.

### 2:28 Surgical Outcome—Case 1: Papillary Craniopharyngioma.

The preoperative bitemporal hemianopsia improved postoperatively with no new neurological deficits. Endocrine outcome was favorable, with no permanent arginine vasopressin deficiency, though hypothyroidism developed. Pathology confirmed papillary craniopharyngioma, BRAF V600E-positive.

### 2:46 Postoperative MRI—Case 1: Papillary Craniopharyngioma.

Near-total resection was confirmed on the MRI postoperative day 2, with a small residual enhancing lesion in the floor of the third ventricle and the pituitary stalk preserved.

### 2:57 Clinical Presentation—Case 2: Adamantinomatous Craniopharyngioma.

The second case is a 37-year-old female who presented with progressive left visual loss and headaches. Neurological exam revealed severe vision impairment in the left side and diffuse left visual field involvement. Endocrine workup returned normal.

### 3:13 Neuroimaging Findings—Case 2: Adamantinomatous Craniopharyngioma.

Imaging revealed an infundibulo-tuberal craniopharyngioma with the stalk not clearly visible. The tumor displayed a multilobulated, cystic-solid heterogeneous architecture with extensive calcifications on CT, characteristic of ACP. MRI demonstrated content consistent with machine oil fluid and heterogeneous enhancement.

### 3:32 Surgical Video—Case 2: Adamantinomatous Craniopharyngioma.

In the second case, following dural opening, the tumor is immediately visualized and a Raman spectroscopy measurement is performed in situ prior to resection. The cyst is then entered, revealing the characteristic machine oil fluid and gross calcifications, consistent with the adamantinomatous subtype. Dissection proceeds along the tumor margins, alternating between internal debulking and capsule dissection to progressively mobilize the lesion. As resection advances, the left optic nerve and the anterior communicating artery complex come into view, guiding the limits of dissection. The remaining tumor tissue is carefully dissected and removed.

In this case, the pituitary stalk was difficult to identify on preoperative imaging, but identified and preserved during surgery. Residual calcifications are addressed and removed where safely accessible.

Reconstruction follows the same multilayer technique as the first case.

### 4:56 Surgical Outcome—Case 2: Adamantinomatous Craniopharyngioma.

Diffuse left visual field involvement improved postoperatively, with no new neurological deficits. Endocrine outcome included permanent arginine vasopressin deficiency and temporary corticotroph deficiency. Pathology confirmed adamantinomatous craniopharyngioma with CTNNB1 mutation, nuclear β-catenin positivity, and negative BRAF V600E.

### 5:16 Postoperative MRI—Case 2: Adamantinomatous Craniopharyngioma.

Near-total resection was achieved, with a small residual adherent to the right optic nerve confirmed on postoperative day 2 MRI.

### 5:30 Risks and Benefits: Endoscopic Endonasal Approach.

The endonasal corridor was used and preferred in both cases because the long axis of the tumor was in line with the endonasal corridor. The major advantage of the endoscopic approach is to avoid brain retraction and reduce optic apparatus manipulation, while allowing direct visualization of the tumor base. It offers superior visual outcomes compared to transcranial approaches.^1^

Risks include CSF leak, meningitis, rare vascular injury, and limited visualization of lateral and upper extensions.^2^ Regardless of approach, resection risks panhypopituitarism and arginine vasopressin deficiency.

### 6:00 Discussion.

We will now compare PCP and ACP across key diagnostic and therapeutic aspects.

### 6:05 Neuroimaging Differences: Classical MRI/CT Features.

PCP and ACP display distinct neuroimaging profiles. PCP affects adults and typically presents as a round, solid or cystic cauliflower lesion located in the third ventricle, with absent calcifications on CT. They show homogeneous gadolinium enhancement.

ACP appears multilobulated with mixed cystic-solid heterogeneous architecture and often carries extensive calcifications. The pituitary stalk is often amputated or not visible.

Neuroimaging alone has limited diagnostic accuracy for subtyping craniopharyngioma. Notably, calcifications—an atypical finding for PCP—were present in this case, underscoring imaging’s limited reliability for subtype determination.^3^

### 6:45 CSF Liquid Biopsy for Preoperative Molecular Diagnosis.

CSF liquid biopsy is emerging as a tool for preoperative molecular diagnosis of craniopharyngioma. Brastianos et al. in 2023 detected circulating BRAF V600E ctDNA in blood and CSF of PCP patients, though sensitivity and limited availability remain constraints.^4^

### 7:02 Differences in Adjuvant Therapeutics: PCP vs ACP.

Arguably, partial resection followed by adjuvant radiotherapy yields equivalent PFS and OS compared to gross-total resection, with no difference between PCP and ACP.^5^

However, for PCP, BRAF/MEK inhibitors have proven transformative, potentially enabling tumor debulking prior to surgery or radiotherapy.

### 7:20 Preoperative and Intraoperative Subtype Determination.

Knowing the craniopharyngioma subtype is therefore critical in determining the aggressiveness of resection, given that alternative systemic therapies are available for PCP.

Despite advances in preoperative characterization, no single tool reliably confirms craniopharyngioma subtype in real time.

### 7:37 Raman Spectroscopy: Background and Rationale for Craniopharyngioma.

We propose translating a Raman spectroscopy endonasal probe—previously validated in pituitary adenoma surgery—into a real-time intraoperative tool for craniopharyngioma subtype discrimination.^6^ The probe uses near-infrared (NIR) laser excitation to generate molecular vibrational fingerprints of tissue biochemistry. It is fiber optic, compatible with the endonasal corridor, and delivers real-time readout in under 3 seconds per acquisition.^7,8^

The rationale for extension to craniopharyngioma rests on distinct biochemical signatures: ACP-specific calcium hydroxyapatite Raman peaks and cholesterol signatures from machine oil cyst fluid are absent in PCP.

### 8:15 Distinct Raman Signature Observed in ACP Tumor.

Here, we first compared the overall Raman spectral profiles acquired from representative ACP and PCP tumor samples (Fig. 1).

Raman profiling revealed a distinct approximately 960 per centimeter peak in ACP, absent in PCP, likely reflecting its characteristic calcified regions.^9^

### 8:31 Hypothesized Molecular Origins of Differential Raman Regions.

Based on peak positions reported in the literature, we generated hypothesis-driven biochemical interpretations for each spectral interval contributing to group separation.^10^

Notably, PCP demonstrated consistently higher normalized Raman intensity across all selected regions, potentially reflecting its more homogeneous and viable epithelial architecture.

The selected regions may capture differences in composition between the two subtypes.

### 8:55 Limitations.

This analysis was performed on a limited number of representative cases.

Also, these results have not undergone peer review in the context of craniopharyngiomas. Only ex vivo data acquired immediately after sampling in the OR is shown. In vivo intraoperative acquisitions were also obtained; however, as these remain under validation, they were not included in the present analysis.

### 9:14 Future Perspectives.

Future studies will therefore focus on validation in larger independent cohorts, histopathology-correlated Raman mapping, multimodal Raman-fluorescence integration, and ultimately the development of real-time intraoperative subtype discrimination approaches.

### 9:29 Conclusions.

PCP and ACP are molecularly and clinically distinct entities.

BRAF/MEK inhibitors have transformed PCP management, offering an alternative therapeutic option.

In the era of precision medicine, craniopharyngioma subtype should inform the aggressiveness of resection.

Preoperative imaging remains valuable for subtype identification, complemented by emerging noninvasive approaches such as CSF liquid biopsy and, potentially, real-time intraoperative Raman-fluorescence spectroscopy, though these preliminary findings require further validation before clinical use.

## Acknowledgments

Artificial intelligence tools were used to assist in the editing of the written text (Anthropic Claude Opus version 4.8), presentation formatting (Anthropic Claude Opus version 4.8), and voiceover (ElevenLabs). We reviewed, verified, and approved all clinical content, surgical decisions, and scientific conclusions.

## Disclosures

N. Nassiri reported grants from the Natural Sciences and Engineering Research Council of Canada (NSERC) outside the submitted work.

## Author Contributions

Primary surgeon: Labidi. Assistant surgeon: Lavigne. Editing and drafting the video and abstract: Labidi, Jemel, Bokasova. Critically revising the work: Labidi, Bokasova, Nassiri, Lavigne, Leblond. Reviewed submitted version of the work: Labidi, Lavigne. Approved the final version of the work on behalf of all authors: Labidi. Supervision: Labidi, Lavigne. Data acquisition: Nassiri.

## Supplemental Information

### Patient Informed Consent

The necessary patient informed consent was obtained in this study.
